# Spontaneous Regression of Plasmablastic Lymphoma in an Immunocompetent Patient: Case Report and Review of the Literature

**DOI:** 10.1155/2022/1142049

**Published:** 2022-05-30

**Authors:** Kee Tat Lee, Nurul Akmar Misron, Norasmidar Abdul Aziz, Chin Hau Wong, Hong Keng Liew

**Affiliations:** ^1^Department of Medicine, Hospital Sultanah Bahiyah, Langgar, Kedah, Malaysia; ^2^Department of Pathology, Hospital Sultanah Bahiyah, Langgar, Kedah, Malaysia

## Abstract

Plasmablastic lymphoma (PBL) is a rare and highly aggressive type of lymphoma, which is commonly associated with human immunodeficiency virus (HIV) infection. Spontaneous regression of aggressive lymphomas is rare as they typically require administration of chemotherapy and radiotherapy for treatment. Here, we describe a case of a spontaneous regression of PBL after nasal biopsy and computed tomography (CT) guided biopsy of paravertebral mass in an immunocompetent patient. We postulate that the patient's immune system may be activated as a result of the stress and physical trauma brought on by nasal and paravertebral mass biopsy. Our case highlights the rare phenomenon of spontaneous regression of lymphoma which needs to be further studied on to establish its underlying pathophysiology.

## 1. Introduction

Plasmablastic lymphoma is a rare and aggressive B cell lymphoproliferative disorder. Delecluse et al. first described this rare disease in the oral cavity of patients with HIV in 1997 [1]. Since the original report, PBL has been more widely recognized and has been incorporated as a new entity in the latest World Health Organization (WHO) classification of lymphoma [2].

However, the diagnosis of PBL remains a diagnostic challenge due to its overlapping features between lymphoma and myeloma. PBL is a high-grade B cell lymphoma that expresses plasma cell markers (CD38, CD138, CD79a, and MUM-1), but typically tests negative for B cell markers (CD19, CD20, and PAX-5). The Ki-67 proliferation index of these tumours is usually high [3].

Despite the advancement in the therapy of aggressive lymphoma, the prognosis of patients with PBL remains dismal with short overall survival. Due to its rarity, it is difficult to establish a standard of care for this disease. Several case reports and case studies show that spontaneous regression can sometimes occur in low-grade lymphomas, but rarely in cases of aggressive lymphomas. Here, we report a case of a 61-year-old gentleman with PBL that spontaneously regressed after CT-guided biopsy of paravertebral mass. Relevant literature is reviewed.

## 2. Case Report

**Figure 1:**
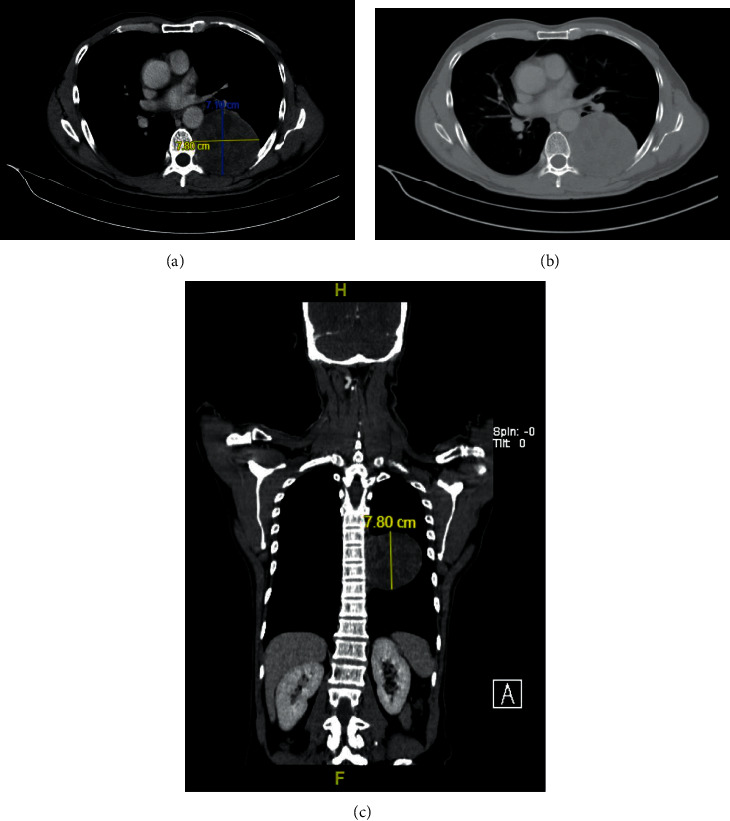
CT showing a well-defined heterogeneously enhancing mass at the left paravertebral region with extension into left T7/8 neural foramina. (a)-(b) Axial view; (c) coronal view.

**Figure 2:**
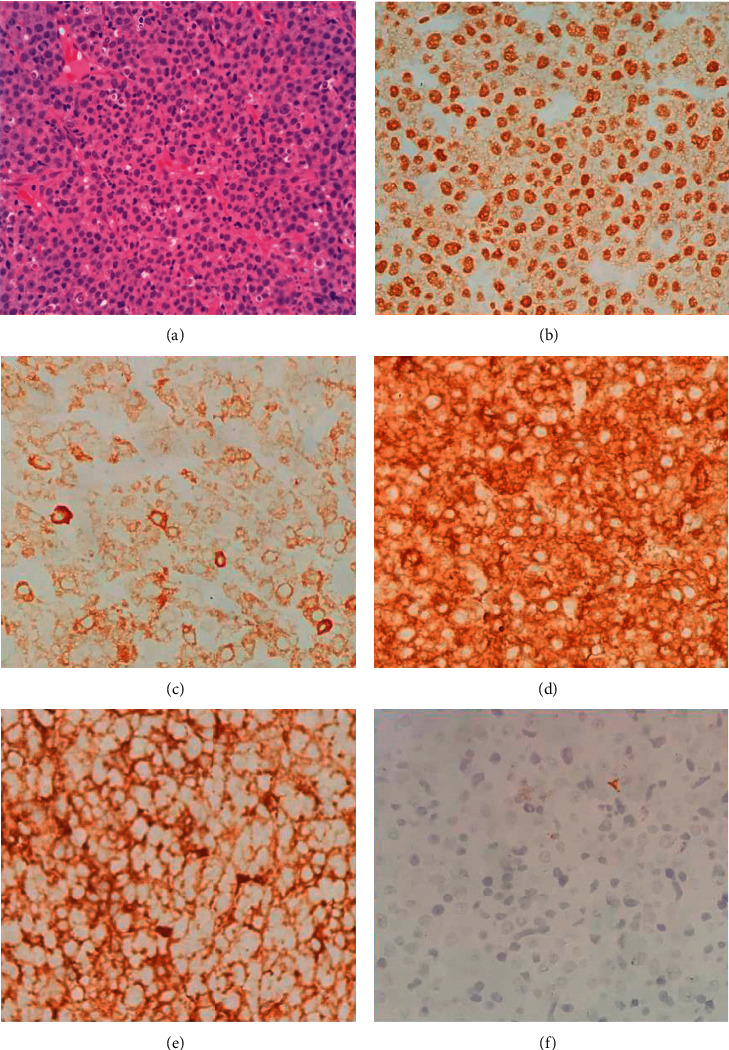
Nasopharyngeal mass biopsy. Hematoxylin and eosin staining ((a) original magnification ×40) and immunohistochemical staining were positive for MUM-1 ((b) original magnification ×40), CD79a ((c) original magnification ×40), kappa ((d) original magnification ×40), and lambda ((e) original magnification ×40) and negative for CD20 ((f) original magnification ×40).

**Figure 3:**
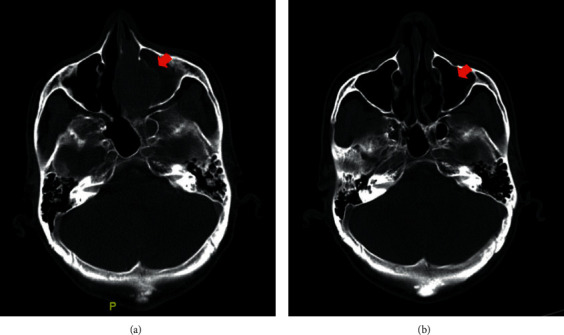
Initial CT showing a mass over left frontal and ethmoid sinuses which occupied almost the entire left nasal cavity (a). Repeated CT showing regression of left nasal mass (b).

A 61-year-old gentleman with no significant medical history presented with the three weeks history of intermittent epistaxis, nasal block, and loss of weight. Physical examination was unremarkable, and he did not have lymphadenopathy or hepatosplenomegaly. Rigid nasal endoscopy revealed a smooth surface mass covering the entire left nasal cavity, pushing the nasal septum to the right and bleeds easily on contact. Subsequently, a CT scan of the paranasal sinus, neck, and thorax revealed a mass over left frontal and ethmoid sinuses which occupied almost the entire left nasal cavity. Incidentally, there was a well-defined heterogeneously enhancing mass at the left paravertebral region (7.1 × 7.8 × 7.8 cm) with extension into left T7/8 neural foramina (Figure 1). Initial laboratory investigation showed white blood cell (WBC) count of 14.2 × 10^3^/uL, hemoglobin (Hb) of 13.8 g/dL, and platelet count of 377 × 10^3^/uL, with normal renal and hepatic function. His lactate dehydrogenase (LDH) was 141 U/L (upper limit of normal: 246). Hepatitis B, hepatitis C, and HIV serologies were nonreactive. Nasopharyngeal mass biopsy was performed, and histological examination highlighted diffuse infiltration by the atypical medium to large lymphoid cells and exhibited multiple membrane bound nucleoli. Immunohistochemistry (IHC) showed that the neoplastic cells were diffusely positive for CD79a, MUM-1, and CD56 and focally positive for PAX-5 (Figure 2). These cells were negative for CD20, CD30, ALK-1, CD3, CD5, CD10, Bcl-2, Bcl-6, CD138, HMB45, and S-100. Nuclear proliferation as assessed by Ki-67 was almost 100%. Unfortunately, Epstein-Barr virus (EBV) encoded RNA by IHC was not performed. Based on the morphological and IHC characteristics, diagnosis of PBL was made. Bone marrow biopsy did not show any marrow infiltration. CT-guided biopsy of the paraveterebral mass was done to rule out possible neurogenic tumour as PBL has rarely been associated with a paravertebral mass. However, the results were inconclusive due to the inadequate sample. He was scheduled for a repeated CT-guided biopsy of the paravertebral mass, and his histopathological examination results came back later which revealed ancient schwannoma. Surprisingly, he reported resolution of his symptoms after the first biopsy of the paravertebral mass, and hence, treatment for PBL was not initiated. Spontaneous regression of his PBL was further confirmed by a repeat rigid nasal endoscopy, which showed good patency of both nasal cavities, and a repeat CT scan of the paranasal sinuses, which revealed spontaneous regression of his left nasal mass (Figure 3). A watch-and-wait approach was employed, and he was followed up closely to look for any evidence of recurrence. He remained in remission during his last follow up after 4 years from diagnosis.

## 3. Discussion

PBL is an aggressive lymphoma which is strongly associated with immunosuppression. In the past decade, there are increasing numbers of PBL case report or series being published worldwide. Some of the reported cases include immunocompetent patients and involve extraoral sites [4–9]. PBL is found predominantly in males (75%), and median age of diagnosis is 50 years old [10]. HIV-negative PBL affects relatively older patients with median age of 71 years as compared to 46 years old in HIV-positive patients [5].

The prognosis of patients with PBL is poor with median overall survival (OS) of 8–15 months [3, 4]. There is no clear concensus on the optimal treatment of PBL. However, chemotherapy such as CHOP (cyclophosphamide, hydroxydaunorubicin, vincristine, and prednisone) or more aggressive chemotherapy regimens such as DA-EPOCH (dose-adjusted etoposide, prednisone, vincristine, cyclophosphamide, and doxorubicin), CODOX-M/IVAC (cyclophosphamide, vincristine, doxorubicin, high-dose methotrexate/ifosfamide, etoposide, and high-dose cytarabine), or hyper-CVAD (hyperfractionated cyclophosphamide, vincristine, doxorubicin, and dexamethasone alternating with methotrexate and cytarabine) have been used with various success rates. Currently, there is no evidence of significant survival benefits with intensive chemotherapy over CHOP regimes [3, 4, 10].

Spontaneous regression of aggressive lymphoma is very uncommon and has rarely been reported in literature. Here, we report a case of sinonasal PBL that spontaneously regressed without chemotherapy. The exact mechanism of spontaneous regression remains unknown, but several hypotheses have been proposed previously. Immune stimulation secondary to active infection is one of the proposed mechanisms that can lead to tumour regression. This was first described by Dr. William Coley 100 years ago, where he reported spontaneous regression of inoperable sarcoma after being infected with *Streptococcus pyogenes* erysipelas [11]. Other proposed hypotheses include the augmented host immune response via humoral and cellular effector mechanisms, which can lead to tumour regression [12].

In this case, our patient reported resolution of his symptoms after biopsy of the paravertebral mass. We postulate that the patient's immune system may be activated as a result of the stress and physical trauma brought on by nasal and paravertebral mass biopsy. This was consistent with findings of several case reports where aggressive lymphomas undergo spontaneous remission after tumour biopsy, suggesting that biopsy might stimulate an antitumour immunity [13–15].

In addition to our present case, we identified and reviewed another eight similar cases of PBL with spontaneous regression (Table 1) [16–23]. The median age of the study cohort was 69 years old (range 35–80). Most of the patients were in Ann Arbor stage I (89%), and only one of them had nodal involvement. Three of them were HIV positive, and PBL regressed after highly active antiretroviral therapy (HAART), which may be related to immune restoration secondary to HAART. In particular, three of the previously reported cases (cases 4, 5, and 8) of PBL were similar to our case, all of them were above 60 years old without significant medical illness, and PBL regressed without chemotherapy or specific treatment.

## 4. Conclusion

Our case highlights the rare phenomenon of spontaneous regression of PBL in an elderly patient. The exact mechanism of this phenomenon remains unknown. However, we postulate that this could be triggered by activation of his immune system after nasal and paravertebral biopsy. Further research is needed to help better understand the pathophysiology behind this phenomenon as this could potentially have major therapeutic implications.

## Figures and Tables

**Table 1:** Summary of clinical characteristic of spontaneous regression of PBL cases without antineoplastic therapy.

No.	Age/sex	Immune status	B symptom	Stage^a^	Primary site	Nodal	Therapy	Follow up	Recurrence
1 [16]	35/male	Yes/HIV	No	I	Maxilla	No	HAART	1 month	No
2 [17]	55/female	Yes/HIV	N/A	N/A	Oral cavity	N/A	HAART	10 months	No
3 [18]	78/female	Yes/MTX treatment	N/A	II	Buccal mucosa	Yes (cervical)	Decrease dose of MTX	2 years	No
4 [19]	80/male	No	No	I	Maxilla	No	No	5 months	No
5 [20]	80/male	No	No	I	Gingiva	No	No	N/A	No
6 [21]	66/female	Yes/HIV	No	I	Maxilla	No	HAART	12 months	No
7 [22]	69/male	Yes/MTX^b^ treatment	No	IV	N/A^c^	No	Stop MTX	12 weeks	No
8 [23]	69/male	No	No	I	Gingiva	N/A	No	2 years	No
9	61/male	No	No	I	Nasal	No	No	4 years	No

^a^Assessed using Ann Arbor staging. ^b^MTX, methotrexate. ^c^N/A, not available.
